# Adaptation and Evaluation of the *WillTry* Tool Among Children in Guam

**DOI:** 10.5888/PCD11.140032

**Published:** 2014-08-21

**Authors:** Tanisha F. Aflague, Rachael T. Leon Guerrero, Carol J. Boushey

**Affiliations:** Author Affiliations: Rachael T. Leon Guerrero, University of Guam, Mangilao, Guam; Carol J. Boushey, University of Hawaii Cancer Center Honolulu, Hawai’i.

## Abstract

**Introduction:**

Fruit and vegetable consumption may reduce risk for chronic disease and obesity. Children’s fruit and vegetable intake is mediated by a preference or willingness to try them. This study’s primary objective was to adapt the previously validated *WillTry* tool and to evaluate the adapted version among children in Guam.

**Methods:**

Adaptations to the *WillTry* tool included both novel fruits and vegetables unique to Guam and common ones. Children aged 3 to 11 years who attended 2 community-based summer day camps in 2013 were shown images matching 14 food questions in an initial interview and in a second interview conducted 3 to 72 hours later. Responses were “no,” “maybe,” or “yes” and were coded as 1, 2, or 3, respectively. A higher score indicated more willingness to try fruits and vegetables. Factor analyses determined components of willingness. Psychometric properties and reliability were analyzed.

**Results:**

Sixty-five children completed the first interview, and 64 completed the second. Factor analyses revealed 3 components (scales):1) local novel (guava, breadfruit, eggplant, sweet sop, star apple, taro leaves), 2) local common (carrot, papaya, long beans, salad greens), and 3) imported (apple, canned peaches, canned corn). All but the imported scale had sufficient internal consistency (Cronbach’s α > 0.69). Each scale had substantial reliability (ICC > 0.76). We found no significant differences by age, sex, or type of camp for any scale. Mean scores were 2.1 (local novel), 2.4 (local common), and 2.7 (imported), and all were significantly different.

**Conclusion:**

The adapted *WillTry* was culturally relevant and had psychometric properties similar to those of the original. An unexpected finding was the tool’s potential for documenting the nutrition transition.

## Introduction

Childhood obesity is increasing in all ethnic and racial groups with greater prevalence in most nonwhite populations (1). Differences in prevalence of childhood obesity among racial groups are complex, likely involving physiology, culture, environment (eg, diet), and interactions among these factors (1,2). Overweight and obese children are at risk for serious chronic illnesses (1). In the US Affiliated Pacific Islands (USAPI), including Guam, a state of emergency was declared in 2010 related to the high prevalence of chronic health conditions in both adults and children in these island communities (3). The childhood overweight and obesity prevalence in Guam in 2011 among children aged 5 to 18 years was 38.5% (4) and was similar to US prevalence rates from the National Health and Nutrition Examination Survey 2009–2010 among non-Hispanic black (39.1%), Hispanic (39.1%), and Mexican American (39.4%) children of similar age ranges (5).

The high prevalence of overweight and obesity reflects a shift in energy balance; energy intake has increased as diets have departed from traditional food systems (ie, diets high in plant-based foods) (6–8). Throughout the USAPI, fruits and vegetables were a prominent part of traditional diets (6,7). The USAPI has a complex legacy of legal and political associations derived from colonialism (9). In pursuit of trade and strategic interest, most of the territorial acquisitions in the Pacific influenced the cultures, foods, and customs of the indigenous peoples. This is also true for Guam, a US territory located in the northwestern Pacific region of Micronesia. The diverse population comprises 37.3% Chamorros, 26.2% Filipinos, 11.5% other Micronesians, 7.0% whites, 6.0% other Asians, and 12.0% other races or people of mixed race/ethnicity (10). Chamorros, the indigenous people of Guam, have changed from an agrarian society to a service and information society, leading to dietary changes (11,12).

Data on food intake of children in Guam, although limited, is comparable to that of children of other ethnic groups. Pobocik et al (6) found that diets of fifth-grade children in Guam were low in fruits and vegetables and high in excess energy, fat, and sugar. Fruit and vegetable intake among children in Guam and the island overall are reported to be lower than recommendations outlined in Healthy People 2010 and *Dietary Guidelines for Americans, 2010* (6,13–15). A large body of evidence suggests many health benefits from a diet high in fruits and vegetables, and some studies show an association with lower risk of overweight in children (13,15). Because inadequate intake of fruits and vegetables has been associated with childhood overweight and obesity (16), promoting their consumption may be effective for preventing overweight and obesity among children on Guam (13).

Preference is one of the key determinants of fruit and vegetable consumption among children that is best supported by evidence (17). Children’s preference or willingness to try fruits and vegetables appears to be an important mediator in predicting their consumption. *WillTry* is a psychometric tool designed to measure children’s self-reported willingness to try fruits and vegetables, both novel and common (18). *WillTry* was originally designed so that its food items could be adapted to test specific foods of interest. The primary objective of this study was to determine if the *WillTry* tool, previously validated in rural, southern US children aged 5 to 14 years (18), could be adapted and validated for use among children aged 3 to 11 years in Guam. In the event the tool was determined to be valid for this population, our additional objective would be to assess whether differences in willingness to try fruits and vegetables varied by summer camp, sex, and age.

## Methods

The limited literature documenting fruit and vegetable consumption in Guam guided the adaptation of the 14 specific food item questions in the *WillTry* tool (Table 1), which are questions 4–17 in Table 1 (2,6,7,19). The original *WillTry*’s question 4 is mixed dish, which was adapted to include commonly consumed mixed dishes on Guam, called “local dish.” Novel fruits and vegetables (ie, guava, breadfruit, eggplant, papaya, star apple, sweet sop, and taro leaves) and common fruits and vegetables (ie, local dishes, apple, carrot, canned peaches, long beans, canned corn, and salad greens) fruits and vegetables consumed in Guam were substituted for the ones in the original *WillTry* to create a version adapted for Guam.

**Table 1:** Results of Interview Using Adapted *WillTry* Tool^a^ Administered to Children Aged 3 to11 Years in Guam at Test (N = 65) and Retest (N = 64)

Question	Yes		Maybe		No	
	Test	Retest	Test	Retest	Test	Retest
There are 3 possible responses for the following question. Please answer “Yes,” “Maybe,” or “No.”						
1. Would you be willing to taste a new food if offered?	40	41	21	19	4	3
The following questions refer to where you might be willing to taste a new food. Please, answer “Yes,” “Maybe,” or “No.”						
a. At home?	42	41	18	17	5	6
b. At a relative’s home?	38	33	16	19	11	12
c. At a restaurant?	43	44	11	15	11	5
d. At school?	42	38	10	14	13	12
There are 3 possible responses for the following questions. Please, answer “Yes,” “Maybe,” or “No.”						
2. Would you be willing to taste a new vegetable?	38	42	16	8	11	4
3. Would you be willing to taste a new fruit?	59	51	5	9	1	4
The following questions will be supported by flash card images – questions 4 – 17. Please, answer “Yes,” “Maybe,” or “No.”						
4. Would you be willing to taste a new dish: eg, eskabeche, tinaktak, kadu?	26	32	21	16	18	16
5. Would you be willing to taste a guava?	45	38	7	12	13	14
6. Would you be willing to taste an apple?	61	61	1	1	3	2
7. Would you be willing to taste breadfruit?	30	28	7	10	28	26
8. Would you be willing to taste a carrot?	53	50	3	3	9	11
9. Would you be willing to taste an eggplant?	21	21	9	7	35	36
10. Would you be willing to taste canned peaches?	44	44	7	7	14	13
11. Would you be willing to taste a papaya?	38	41	7	7	20	16
12. Would you be willing to taste long beans?	36	35	2	5	27	24
13. Would you be willing to taste canned corn?	54	53	3	5	8	6
14. Would you be willing to taste a star apple?	45	46	6	7	14	11
15. Would you be willing to taste salad (greens)?	42	43	8	6	15	15
16. Would you be willing to taste sweet sop?	22	26	6	3	37	35
17. Would you be willing to taste taro leaves?	27	24	11	16	27	24
Three responses are provided for the following 2 questions.						
18. Which of these best describes you?	Eat only favorite foods		Eat most foods		Eat any food offered		
	14	29	19	22	32	13
19. Which of these best describes your parent?	41	33	9	20	11	9

a The adaptation was the incorporation of fruits and vegetables common to Guam either locally produced or imported, locally produced and uncommon, and local mixed dishes.

Data were collected from children registered in existing summer day camps in Guam during 2013. Children were recruited from 2 camps: a cultural immersion summer program with children aged 3 to 11 and a university-based recreational sports camp with children aged 5 to 15. In the latter camp, only children aged 5 to 8 were recruited for this study. Each camp had full schedules of structured activities and scheduled down time. Both summer programs were open to children of all ethnic groups. During drop-off or pick-up, parents were asked whether their children could participate in the study. Informed consent was obtained from the parents and assent from their children. One parent from the sports camp declined the invitation for her child to participate. The Human Studies Program of the University of Hawaii and the University of Guam Institutional Review Board approved study methods.

Parents completed information about the characteristics of the child relevant to the administration of the adapted *WillTry*, including date of birth, sex, ethnicity, language spoken, and religion. The adapted *WillTry* tool was interview-administered to all children recruited at both summer camps. Researchers conducted one-on-one interviews with the children following a scripted questionnaire. Interviews were conducted in English except when referring to Chamorro names of local foods. For the 14 specific food-item questions, participants were shown corresponding color images of each food. The images showed how the fruits and vegetables were usually prepared and consumed in Guam. All children were given an option to complete the interview a second time. The second interviews were administered approximately 3 to 72 hours after the first administration. For participation and cooperation, participants received a $5 gift card after the first administration of the adapted *WillTry*. If participants completed the interview a second time, they received another $5 gift card.

Data were entered by using a Microsoft Access (Microsoft Corp) tool specifically designed for this study. Double-data entry procedures were used, and the procedure, PROC COMPARE, in SAS 9.3 (SAS Institute, Inc) was performed until both data entries achieved 100% matching. To determine the food scales represented by the adapted *WillTry* tool, data reduction methods from the original *WillTry* (18) were repeated. Factor analyses were run to determine whether the adapted *WillTry* items represented a single dimension or sources of variance representing different characteristics of willingness to try fruits and vegetables. The responses for willingness to try were designated as 1 = no, 2 = maybe, and 3 = yes. Each scale was calculated as the total of the responses for each item in the scale, and each score was calculated as the scale divided by the number of items in the scale. Variables were examined for meeting the assumptions of a normal distribution, and none needed transformation. Quantitative variables were examined by using means and standard deviations, and frequencies were completed for categorical variables. Pearson correlations were performed to examine the relationships between the scales. Differences in mean responses between the test–retest and scores were examined by using paired *t* tests.

Statistical analyses from the original *WillTry* study (18) were also used to confirm the stability of the psychometric properties of the adapted *WillTry* tool. Cronbach's α-coefficients were used to assess internal consistency, and 2-way random intraclass correlation coefficients (ICCs) were used to assess test–retest reliability. The general guidelines to measure strength of reliability as in the original *WillTry* tool (18) were applied as reliability statistics (ICC < 0, poor; ICC 0–0.20, slight; ICC 0.21–0.40, fair; ICC 0.41–0.60, moderate; ICC 0.61–0.80, substantial; and ICC 0.81–1.00, almost perfect). Unless otherwise noted, all analyses were completed by using SPSS version 21.0.0 (IBM Corporation).

Differences by sex and camp for the adapted *WillTry* scales and scores were compared by independent sample *t* test. Ages were divided by tertiles (ie, 3–5 y, 6, 7 y, 8–11 y). Differences by age for the adapted *WillTry* scales and scores were compared by ANOVA. Multivariate linear regression was used to evaluate whether each scale and score (dependent variables) was associated with sex, camp, or age (independent variables). Statistical significance was set at *P* < .05, and reported *P* values were 2-sided.

## Results

Sixty-five children aged 3 to 11 consented to participate (Table 2). Of the 33 children enrolled in the cultural immersion summer program, all completed the first administration of the adapted *WillTry* tool, and 32 (97%) completed the retest. Of the 32 children enrolled in the university-based sports camp, all completed both the test and retest. Of the 65 children, 40 were girls (3–11 y) and 25 were boys (5–11 y). Parents identified 58 (89%) of the children as Chamorro (32 [49%] as Chamorro only, 26 [40%] as Chamorro and other race/ethnicity), and 7 (11%) as other (3 white [5%], 2 Asian [3%], 1 black [1.5%], and 1 mixed race/ethnicity [1.5%]).

**Table 2:** Characteristics of Children (N = 65) Aged 3 to 11 Years From Summer Camps in Guam Interviewed With the Adapted *WillTry* Tool

Characteristic	Boys n = 25 (38%)	Girls n = 40 (62%)	Total n = 65 (100%)
Type of camp			
Cultural immersion	11	22	33
Sports	14	18	32
Race/ethnicity			
Chamorro only	11	21	32
Chamorro and other race/ethnicity	9	17	26
Other	5	2	7
Age, y (tertiles)			
3–5	3	15	18
6–7	11	16	27
8–11	11	9	20

Factor analysis performed on all the food questions revealed 5 components (food-related questions and specific food items); 2 components accounted for 35% and 10% of the total variance. The foods with the highest factor loadings (>0.40) in the first component were the local foods (ie, breadfruit, eggplant, salad greens, sweet sop, taro leaves, long beans). Next, we restricted factor analysis to 3 components for the specific food items only and removed 1 food (ie, local dish). This resulted in 3 components accounting for 40%, 12%, and 8% of the variance. On the basis of item factor loadings above 0.40 within each component of the latter factor analysis, distinct differences by source (ie, local or imported) and familiarity (ie, novel or common) emerged. From these components, 3 scales of fruits and vegetables were created and designated as local novel (ie, guava, breadfruit, eggplant, star apple, sweet sop, taro leaves), local common (ie, carrot, papaya, long beans, salad greens), and imported (ie, apple, canned peaches, canned corn). The scales were not perfectly correlated using Pearson’s correlation coefficient, showing that each scale measured different constructs with regard to willingness to try (0.668, local novel and common; 0.446, common and imported; and 0.389, imported and local novel).

The local novel scale included 6 items with a total scale range (sum of all possible responses for each scale) of 6 to 18; the local common scale was 4 items with a total scale range of 4 to 12, and the imported food scale was 3 items with a total scale range of 3 to 9. The adapted *WillTry* scores for each food scale ranged from 1 to 3 (total scale divided by the number of items in each scale). We found no significant differences in any scale or any score between the first and second administration of the adapted *WillTry* tool (Table 3).

**Table 3:** Internal Consistency and Reliability Measures for the Adapted *WillTry* Interview Tool Completed by Children Aged 3 to 11 Years on Guam

Scale (Food Type)	Total Scale^a^			Score,^b^ Mean	Cronbach’s α Test	ICC Test–Retest
	Mean	Median	SD			
Test (N = 65)						
Local novel^c^	12.6	12.0	4.00	2.1^f^	0.84	—
Local common^d^	9.5	10.0	2.62	2.4^f^	0.75	—
Imported^e^	8.1	9.0	1.50	2.7^f^	0.60	—
Retest (N = 64)						
Local novel^c^>	12.6	12.0	3.90	2.1^f^	0.83	0.90^g^
Local common^d^	9.6	10.0	2.47	2.4^f^	0.69	0.77^g^
Imported^e^	8.1	9.0	1.32	2.7^f^	0.47	0.84^g^

Abbreviations: SD, standard deviation; ICC, intraclass correlation coefficient (2-way random effects); —, not applicable.

a Total scale is the sum of responses to each specific food question in each scale.

b Score is the total scale mean divided by the number of items in the scale.

c Local novel scale includes guava, breadfruit, eggplant, star apple, sweet sop, and taro leaves (6 items; range 6–18).

d Local common scale includes carrot, papaya, long bean, and salad (4 items; range 4–12)

e Imported scale includes apple, canned peaches, and canned corn (3 items; range 3–9)

f *P* < .001

g Correlation is significant at the *P* ≤ .001 level (2-tailed).

Internal consistency was examined for each of the 3 food scales. All but the imported food scale had sufficient internal consistency (Cronbach’s α ≥ 0.70) at both test and retest (Table 3). All 3 scales had substantial reliability (local novel, ICC 0.90; local common, ICC 0.77; and imported, ICC 0.84) (Table 3). We examined food scales for internal consistency by age tertiles (T) (T1 = 3–5 y, T2 = 6–7 y, and T3 = 8–11 y) by using the first administration of the adapted *WillTry* tool. T1 maintained sufficient internal consistency for all food scales (Cronbach’s α ranging from 0.74 to 0.84, (Table 4). Less than optimal Cronbach’s α values were found in the T3 group for the imported food scale (0.46) and in the T2 group for the imported (0.29) and the local common (0.57) scales (Table 4). For the T2 group, all 3 scales had significant reliability (local novel, ICC 0.93; local common, ICC 0.87; imported, ICC 0.95) T1 and T3 children each had 2 scales within the significant reliability range (Table 4).

**Table 4:** Internal Consistency and Reliability Measures by Age Tertile^a^ for the Adapted *WillTry* Interview Tool Completed by Children Aged 3 to 11 Years in Guam

Scale (Food Type)	Test (N = 65), Cronbach’s α	Test–Retest (N = 64), ICC
Local novel food^b^		
Tertile 1	0.84	0.86^c^
Tertile 2	0.86	0.93^c^
Tertile 3	0.84	0.93^c^
Local common food^d^		
Tertile 1	0.74	0.42
Tertile 2	0.57	0.87^c^
Tertile 3	0.88	0.97^c^
Imported food^e^		
Tertile 1	0.77	0.86^c^
Tertile 2	0.29	0.95^c^
Tertile 3	0.46	0.47

Abbreviations: ICC, intraclass correlation coefficient (2-way random effects).

a Tertile 1: 3 to 5 years; tertile 2: 6 to 7 years; tertile 3: 8 to 11 years.

b Local novel scale includes guava, breadfruit, eggplant, star apple, sweet sop, and taro leaves (6 items; range 6–18).

c Correlation is significant at *P* ≤ .001 (2-tailed).

d Local common scale includes carrot, papaya, long bean, and salad (4 items; range 4–12).

e Imported scale includes apple, canned peaches, and canned corn (3 items; range 3–9).

By using the first administration of the adapted *WillTry* tool, the total mean for the local novel scale was 12.2 for boys and 12.8 for girls; local common scale was 8.9 for boys and 9.9 for girls; and imported scale was 7.9 for boys and 8.2 for girls. The adapted *WillTry* scores for each of the 3 food scales were 2.0, 2.2, and 2.6 for boys and 2.1, 2.5, and 2.7 for girls. The mean scores and scales by camp and age were similar to those by sex. There were no significant differences by age, sex, and camp. The lack of any significant differences by these characteristics remained even after multivariate analyses. However, the overall mean scores for local novel were lowest at 2.1 followed by local common at 2.4 (Table 3), and each was significantly different (*P* < .001). The highest overall mean score, indicating most willing to try, was the imported score at 2.7 and was significantly higher than both local common (*P* < .001) and local novel (*P* < .001).

## Discussion

The adapted *WillTry* tool has psychometric properties similar to the original *WillTry* tool for measuring children’s willingness to try both novel and common fruits and vegetables in terms of factorial structure, internal consistency, and test–retest reliability among younger children within a different population. All but the imported food scale had sufficient internal consistency (Cronbach’s α ≥ 0.70), which may be attributed to the few items in the scale. The substantial reliability for all but the imported food scale and sufficient internal consistency for all scales among the youngest age group (T1) were noteworthy outcomes. The adaptability of the *WillTry* tool permitted the incorporation of novel and common foods of research interest. The adapted *WillTry* tool promises to be a useful adjunct for outcome assessment of programs promoting fruit and vegetable consumption among children in Guam. An unanticipated result of the study was the creation of the 3 distinct food scales that described dietary patterns outlined by the nutrition transition stages (8) as discrete separation of foods by imported and local, and the local food group further delineated by familiarity. The children’s willingness to try novel local foods was surprisingly less than their willingness to try imported foods; this finding is consistent with dependence among island cultures on imported foods (2,6,7,11). Some of the possible avenues through which children have been introduced to imported foods include supplemental food programs such as the Women, Infants, and Children Supplemental Nutrition Program; Commodity Supplemental Food Programs; Child and Adult Care Food Program; and the National School Lunch Program (20,21). These unexpected attributes of the adapted *WillTry* tool offer the potential for monitoring dietary changes and making the tool useful for culturally relevant assessment in the USAPI.

The lack of significant differences in willingness to try fruits and vegetables between sex, camp, and age supports use of the adapted *WillTry* tool to assess the effectiveness of culturally based interventions addressing consumption of fruits and vegetables. Furthermore, the unanticipated emergence of the 3 distinct food scales (local novel, local common, and imported) elucidated a new concept for use of the adapted *WillTry* tool. The 3 scales along with the distinct scores associated with each scale were consistent with the nutrition transition and dietary acculturation processes that highlight the departure from consumption of traditional foods (8,22). Thus, interventions to improve fruit and vegetable consumption among children in Guam should consider incorporating cultural practices and should use the adapted *WillTry* tool, which has the potential to capture shifts in local food preferences in general and in fruits and vegetables in particular.

The specific food items of the adapted *WillTry* were selected from the small number of published studies on foods frequently consumed by children and adults in Guam (6,7) and from culturally relevant modifications to a nutrition curriculum in Guam (19). These resources provided food lists, one of which was useful and relevant, by Pobocik et al (6), indicating that most of the fruits and vegetables reported as frequently consumed by children (10.8% of foods consumed) were processed and that consumption of traditional fruits and vegetables was reported infrequently. Also from this list, the top 3 fruits and vegetables, other than fruit juices, were the same foods that emerged as the unanticipated imported food scale, thus reflecting a considerable reliance on imported foods as early as 1993 and 1994. Defining imported and market foods as being shipped from elsewhere and purchased in a store aids in understanding shifts away from traditional food systems (23,24).

All foods in the local common food scale were also found on the food list reported by Pobocik et al (6), and these foods appeared between the fortieth and sixty-first of 85 most frequently consumed foods listed, hence “common.” For this food scale, the “local” label applies to foods introduced into traditional and local dishes during different pivotal colonial periods that are usually featured at cultural celebrations (25). Fruits and vegetables that made up the local novel scale are foods that have been identified as traditional foods recommended for use in a culturally relevant nutrition curriculum (19) or identified on the Guam Fruits and Vegetables Availability Charts (26). Furthermore, these foods are not listed as common for children (6) and are therefore labeled as novel. Aligning with the process of the nutrition transition, fruits and vegetables found in the local novel food scale may indeed be novel for children despite seasonal local availability.

Several strengths of this study include the use of a previously validated tool associated with actual consumption patterns of fruits and vegetables among children (18). An additional strength was that only 1 child was unavailable to complete the retest; thus, the numbers for the comparison for test–retest were nearly perfect. The youngest children were in early childhood (3–4 y), a group often overlooked because of literacy issues and attention span. The adapted short questionnaire administered by an interviewer performed well with this understudied age group.

The study is not without limitations in that a convenient sample was used and may not be representative of all children in Guam. Nonetheless, the ethnic profile closely matched the ethnic distribution from the 2010 US Guam Census (10), supporting generalizability. The small sample size may have limited the psychometric properties; however, this effect may have been overshadowed by the homogeneity of the sample from Guam, because the psychometric properties’ results were fairly robust. The small number of food items in the imported scale likely influenced the psychometric properties for that scale. Future adaptations of this tool might consider selecting an equal number of imported, local novel, and local common foods if the tool is adapted for other indigenous populations to explore shifts in dietary patterns.

The *WillTry* tool for assessing children’s willingness to try fruits and vegetables was successfully adapted as a culturally relevant tool for use among children in Guam aged 3 and 11 years. An unexpected finding was the tool’s potential for documenting the transition from local to imported foods in children’s diets.

## References

[1] Caprio S , Daniels SR , Drewnowski A , Kaufman FR , Palinkas LA , Rosenbloom AL , Influence of race, ethnicity, and culture on childhood obesity: implications for prevention and treatment: a consensus statement of Shaping America's Health and the Obesity Society. Diabetes Care 2008;31(11):2211–21. 10.2337/dc08-9024 18955718 PMC2571048

[2] World Health Organization Regional Office for the Western Pacific. Diet, food supply, and obesity in the Pacific. Geneva (CH): World Health Organization; 2003.

[3] Pacific Islands Health Officers Association. The burden of non-communicable disease. Board resolution no. 48-01. http://www.pihoa.org/fullsite/newsroom/wp-content/uploads/downloads/2012/06/NCD_Emergency_Declaration.pdf. Accessed July 3, 2014.

[4] Wilken LR , Novotny R , Fialkowski MK , Boushey CJ , Nigg C , Paulino Y , Children’s Healthy Living (CHL) Program for remote underserved minority populations in the Pacific region: rationale and design of a community randomized trial to prevent early childhood obesity. BMC Public Health 2013;13 :944. 10.1186/1471-2458-13-944 24107083 PMC3851862

[5] Ogden CL , Carroll MD , Kit BK , Flegal KM . Prevalence of obesity and trends in body mass index among US children and adolescents, 1999–2010. JAMA 2012;307(5):483–90. 10.1001/jama.2012.40 22253364 PMC6362452

[6] Pobocik RS , Richer JJ , O’Donnel BK . Foods most frequently consumed by fifth grade children on Guam. Pac Health Dialog 1999;6(1):57–64.

[7] Pobocik RS , Trager A , Monsen LM . Dietary patterns and food choices of a population sample of adults on Guam. Asia Pac J Clin Nutr 2008;17(1):94–100. 18364333

[8] Popkin BM . Contemporary nutritional transition: determinants of diet and its impact on body composition. Proc Nutr Soc 2011;70(1):82–91. 10.1017/S0029665110003903 21092363 PMC3029493

[9] Opeskin B , MacDermott T . Resources, population and migration in the Pacific: connecting islands and rim. Asia Pac Viewp 2009;50(3):353–73. 10.1111/j.1467-8373.2009.01407.x

[10] US Census Bureau. Guam State Data Center Bureau of Statistics and Plans. 2010 census of population and housing Guam. Demographic profile summary file; 2012. http://bsp.guam.gov/index.php?option=com_content&view=article&id=166%3A2010-census-demographic-profile-summary-file&catid=1&Itemid=100008. Accessed October 30, 2013.

[11] Hankin J , Reed D , Labarthe D , Nichaman M, Stallones R . Dietary and disease patterns among Micronesians. Am J Clin Nutr 1970;23(3):346–57. 5436641 10.1093/ajcn/23.3.346

[12] Pollock N . Food habits in Guam over 500 years. Pacific Viewpoint 1986;27(2):120–48.

[13] Healthy People 2010. Washington (DC): US Department of Health and Human Services; 2010. http://www.healthypeople.gov/2010/. Accessed February 11, 2013.

[14] Lorson BA , Melgar-Quinonez HR , Taylor CA . Correlates of fruit and vegetable intakes in US children. J Am Diet Assoc 2009;109(3):474–8. 10.1016/j.jada.2008.11.022 19248865

[15] Dietary Guidelines for Americans. 7th edition. Washington (DC): US Department of Agriculture and US Department of Health and Human Services; 2010. http://www.cnpp.usda.gov/DGAs2010-PolicyDocument.htm. Accessed February 11, 2013.

[16] Miller P , Moore RH , Kral TV . Children’s daily fruit and vegetable intake: associations with maternal intake and child weight status. J Nutr Educ Behav 2011;43(5):396–400. 10.1016/j.jneb.2010.10.003 21764642

[17] Rasmussen M , Krolner R , Klepp KI , Lytle L , Brug J , Bere E , Determinants of fruit and vegetable consumption among children and adolescents: a review of the literature. Part I: Quantitative studies. Int J Behav Nutr Phys Act 2006;3:22. 10.1186/1479-5868-3-22 16904006 PMC1564033

[18] Thomson JL , McCabe-Sellers BJ , Strickland E , Lovera D , Nuss HJ , Yadrick K , Development and evaluation of WillTry. An instrument for measuring children’s willingness to try fruits and vegetables. Appetite 2010;54(3):465–72. 10.1016/j.appet.2010.01.012 20116407

[19] Pobocik RS , Montgomery D , Roff Gemlo L . Modification of a school-based nutrition education curriculum to be culturally relevant for Western Pacific Islanders. J Nutr Educ 1998;30(3):164–9. 10.1016/S0022-3182(98)70307-3

[20] Damman S , Eide WB , Kuhnlein HV . Indigenous peoples’ nutrition transition in a right to food perspective. Food Policy 2008;33(2):135–55. 10.1016/j.foodpol.2007.08.002

[21] Popkin BM , Adair LS , Ng SW . Global nutrition transition and the pandemic of obesity in developing countries. Nutr Rev 2012;70(1):3–21. 10.1111/j.1753-4887.2011.00456.x 22221213 PMC3257829

[22] Satia-Abouta J , Patterson R , Neuhouser M , Elder J . Dietary acculturation applications to nutrition research and dietetics. J Am Diet Assoc 2002;102(8):1105–18. 12171455 10.1016/s0002-8223(02)90247-6

[23] Kuhnlein HV , Receveur O , Soueida R , Egeland GM . Arctic indigenous peoples experience the nutrition transition with changing dietary patterns and obesity. J Nutr 2004;134(6):1447–53. 15173410 10.1093/jn/134.6.1447

[24] Snowdon W , Raj A , Reeve E , Guerrero RL , Fesaitu J , Cateine K , Processed foods available in the Pacific Islands. Global Health 2013;9:53. 10.1186/1744-8603-9-53 24160249 PMC4016479

[25] Salas MC , Tolentino DM . Ancient Chamorro food and diet. Guam: Guampedia; 2009. http://guampedia.com/ancient-chamorro-food-and-diet/. Updated January 9, 2013. Accessed October 5, 2013.

[26] Bamba J , McConnell J , Barber B , Cruz JA , Tuquero J , Cruz FJ . Guam fruit and vegetable availability charts. Agriculture and Natural Resources, Guam Cooperative Extension, College of Natural and Applied Sciences, University of Guam and USDA Natural Resources Conservation Services; 2009. http://mysare.sare.org/mySARE/assocfiles/935124fruit%20poster.pdf. Accessed July 9, 2014.

